# Supplementary material for: Ultralight covalent organic framework/graphene aerogels with hierarchical porosity
Source: Nat Commun. 2020 Sep 18;11:4712. doi: 10.1038/s41467-020-18427-3 (PMC7501297; doi:10.1038/s41467-020-18427-3)
Supplement: Supplementary file 1 — Supplementary Information [file 41467_2020_18427_MOESM1_ESM.pdf]

Supplementary Information

Ultralight covalent organic framework/graphene aerogels with hierarchical porosity

Li et al.

Supplementary Methods

Materials.

All chemicals were purchased from commercial sources and used without further treatment. p-Toluenesulfonic acid monohydrate (Sigma-Aldrich, ≥ 98.5%), Diaminoanthraquinone (TCI, > 97.0%), Dimethyl sulfoxide (DMSO) (Carl Roth, ≥ 99.5%), Phenoxin (Sigma-Aldrich, > 99.5%), Methanol (Carl Roth, ≥ 99%), Tetrahydrofuran (Acros Organics, 99+%), Chloroform (Carl Roth, ≥ 99.5%), Silicone oil (Carl Roth), Hexane (Fisher, > 99%), Cyclohexane (Carl Roth, ≥ 99.5%), Toluene (Carl Roth, ≥ 99.5%), Ethyl acetate (Sigma-Aldrich, 99.8%), Acetone (Carl Roth, ≥ 99.5%), N,N-Dimethylformamide (DMF) (Carl Roth, ≥ 99.9%), Ethylene glycol (Sigma-Aldrich, 99.8%), Dioxane (Carl Roth, ≥ 99.8%), Ethanol (Carl Roth, ≥ 99.8%), N,N-Dimethylacetamide (DMA) (Carl Roth, ≥ 99%), Oil Red O (Alfa Aesar).

Supplementary Figures

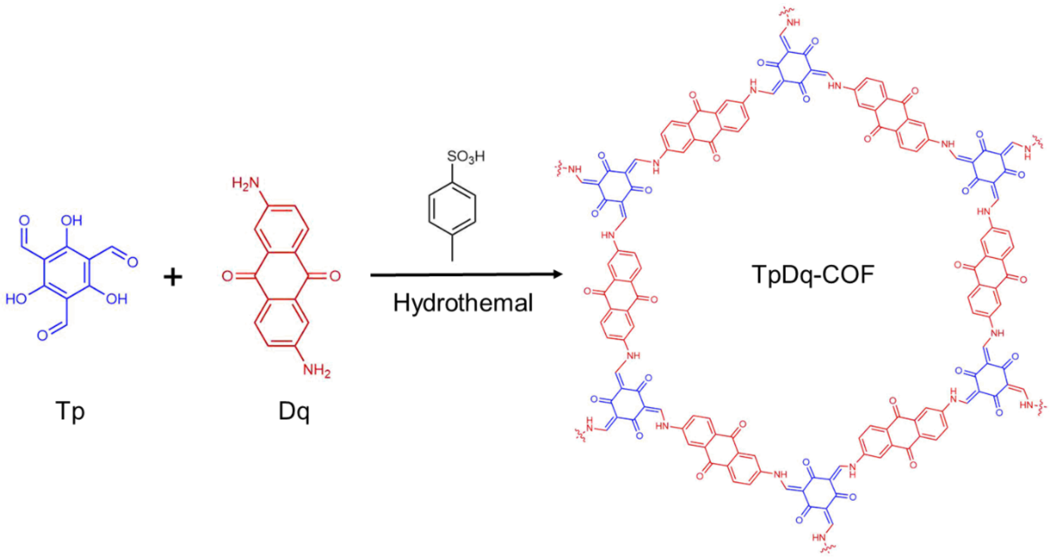

Supplementary Figure 1. Schematic representation of the synthesis of TpDq-COF.

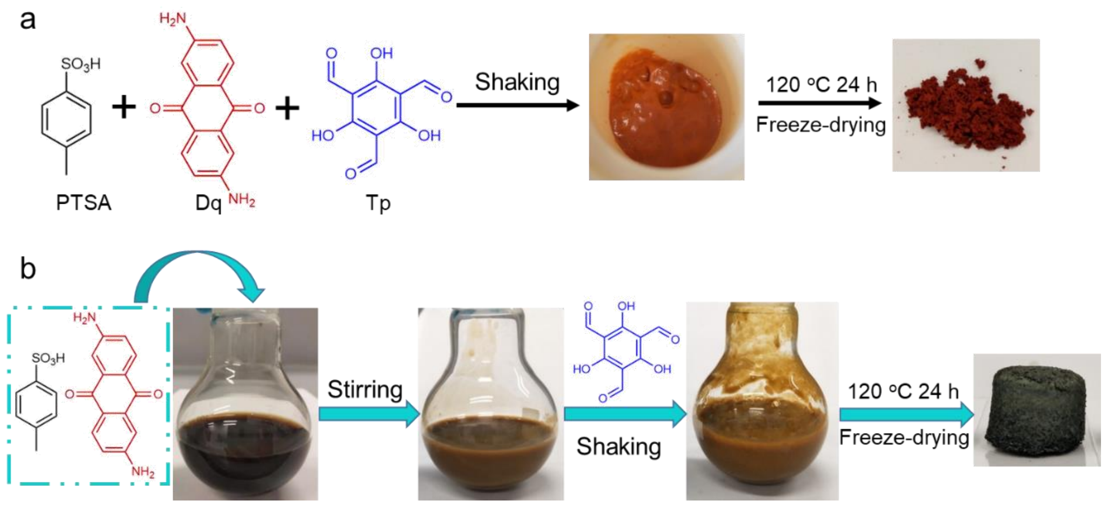

Supplementary Figure 2. Synthesis of (a) TpDq-COF and (b) COF/rGO aerogel *via* the hydrothermal method.

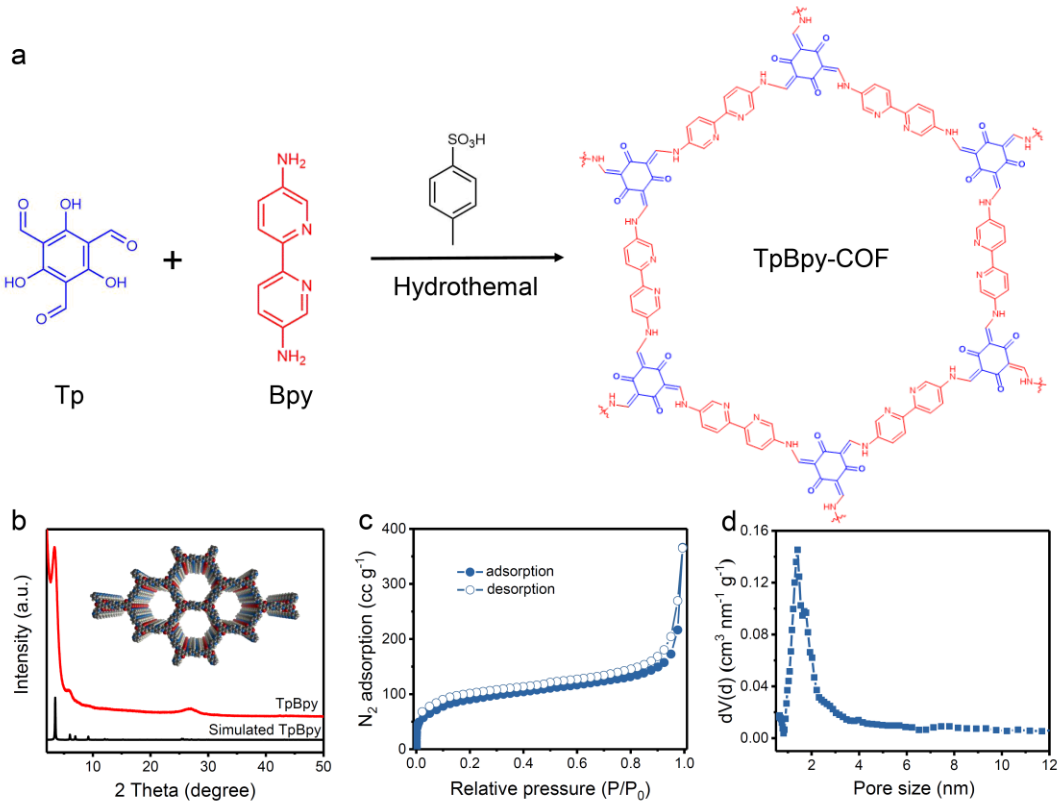

Supplementary Figure 3. (a) Schematic representation of the synthesis of TpBpy-COF. (b) PXRD pattern of TpBpy-COF and simulated XRD pattern from the modelled structure in eclipsed form. The inset shows a space-filling packing model of TpBpy-COF. (c) N_2 adsorption-desorption isotherms and (d) pore size distribution for TpBpy-COF.

Supplementary Note 1

The hydrothermal method is also applicable to TpBpy-COF (Bpy = 2,2′-bipyridine-5,5′-diamine). Well-ground PTSA (59.4 mg, 0.31 mmol), Bpy powder (10.4 mg, 0.056 mmol) and 5 mL of water were mixed thoroughly and shaken well in a vortex shaker for 5 min. Then, 7.8 mg of Tp (0.037 mmol) was added to the yellow solution and shaken for another 20 min. The solution was transferred into an autoclave and heated at 120 °C for 24 h. Then, the obtained solid was sequentially washed with hot water, acetone to remove unreacted reagents and monomer fragments. Finally, the material was filtered, collected and oven-dried at 80 ^{o}C. The obtained

TpBpy-COF display a peak at 3.6° (2θ) corresponding to the reflection from the (100) plane, in good agreement with the corresponding simulated XRD pattern from the modelled structure, confirming the formation of the crystalline structure of TpBpy-COF (Supplementary Fig. 3b). The N_2 adsorption isotherm of TpBpy-COF displays a type I isotherm with a steep increase at low relative pressures, indicating the microporosity of TpBpy, which was further demonstrated calculating the corresponding pore size distribution (Supplementary Fig. 3c-d). TpBpy-COF has a BET surface area of 331 m^2 g^{-1} and a pore volume of 0.344 cm^3 g^{-1}.

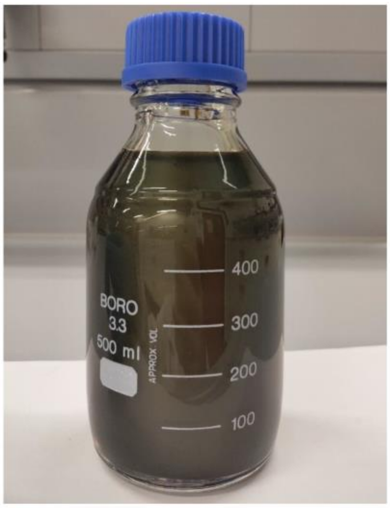

Supplementary Figure 4. Photograph of 5.0 mg mL^{-1} GO solution.

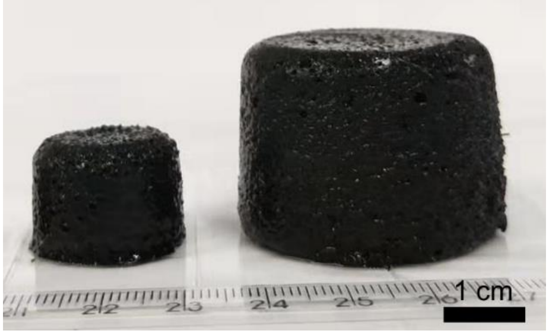

Supplementary Figure 5. Photograph of COF/rGO hydrogels prepared by using a 20 ml (left) and a 120 ml (right) autoclave, respectively.

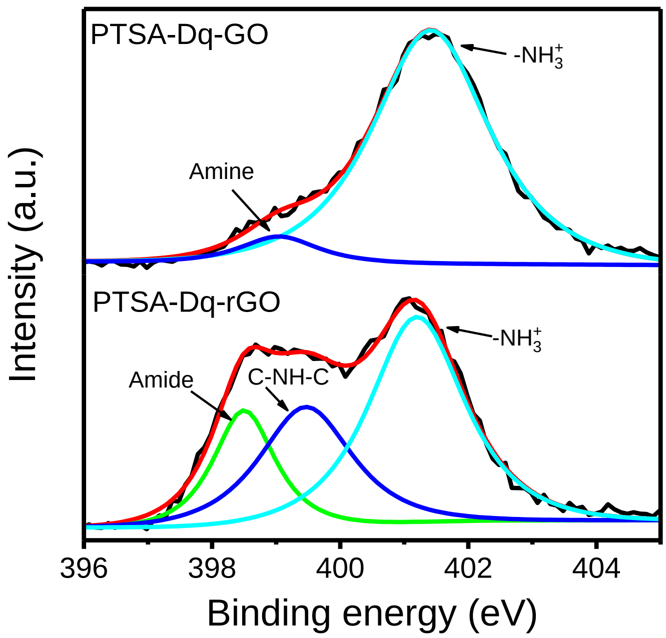

Supplementary Figure 6. N1s XPS spectra of PTSA-Dq-GO and PTSA-Dq-rGO.

Supplementary Note 2

To illustrate the possible interaction between graphene oxide and the monomers in the initial polymerization of the COF, mixtures of the amine-functionalized monomer Dq and GO were prepared and analyzed by XPS at different stages, using reaction conditions comparable to the ones used for COF formation. (1) Preparation of PTSA-Dq-GO: Well-ground PTSA (59.4 mg, 0.31 mmol), Dq (13.4 mg, 0.056 mmol) and 5 mL of water were mixed thoroughly and shaken well in a vortex shaker for 5 min. The yellow solution was added into 4.3 mL of 5 mg mL^{-1} GO dispersion dropwise and stirred for 30 min to obtain a homogeneous dispersion. After freeze-drying, PTSA-Dq-GO was obtained. The high-resolution N 1*s* spectrum of PTSA-Dq-GO shows two components: mainly protonated amine ($-NH_3^+$, 401.4 eV) and some free amine ($-NH_2$, 399.0 eV) groups[1,2]. This is expected when adding PTSA to the amine-functionalized Dq monomer, showing that at this stage interactions between GO and Dq are exclusively electrostatic and/or hydrogen bond interactions.

(2) The PTSA-Dq-GO mixed solution was then transferred into an autoclave and heated at 120 °C for 24 h. Then, the hydrogel was freeze-dried to obtain PTSA-Dq-rGO. The high-resolution N 1*s* spectrum of PTSA-Dq-rGO reveals again the presence of protonated amine (401.2 eV) but also amide (398.5 eV) and C-NH-C (399.5 eV) groups[3], suggesting that covalent grafting of Dq on graphene oxide is possible during the hydrothermal process and thus might be an initial step for growing the COF on the graphene sheet.

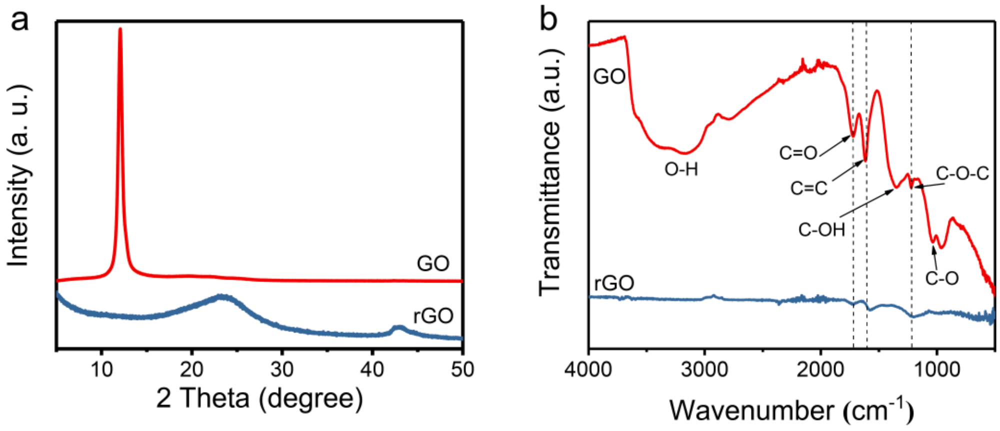

Supplementary Figure 7. (a) XRD patterns and (b) IR spectra of GO and rGO.

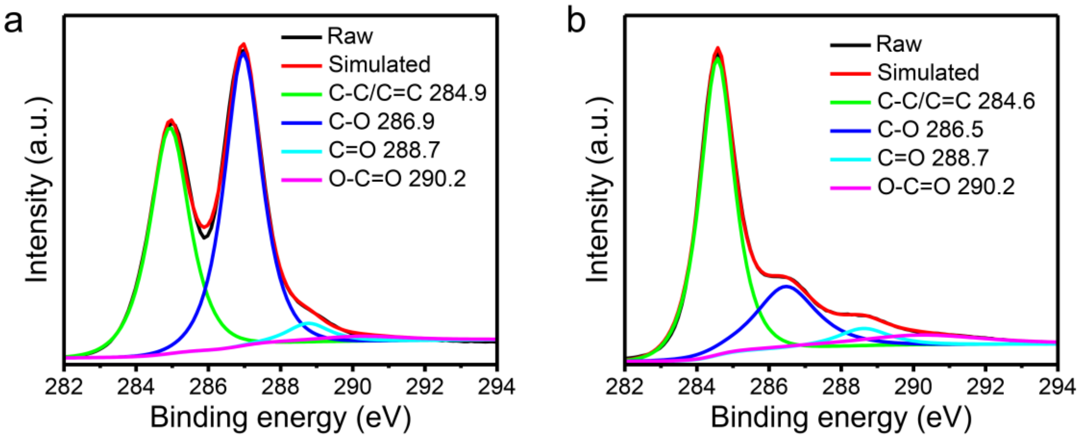

Supplementary Figure 8. C1s XPS spectra of (a) GO and (b) rGO.

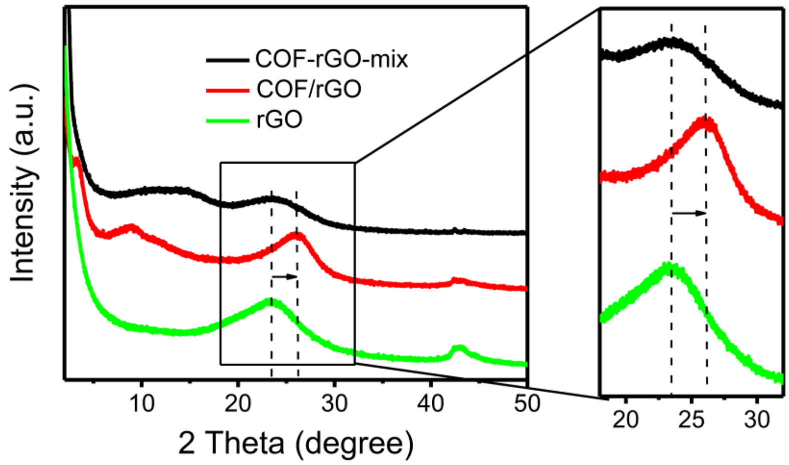

Supplementary Figure 9. PXRD patterns of rGO, COF/rGO and a physical mixture of the pure COF and pure rGO (COF-rGO-mix). The COF-rGO-mix was obtained by mixing and grinding COF and rGO with a mass ratio of 1:1.

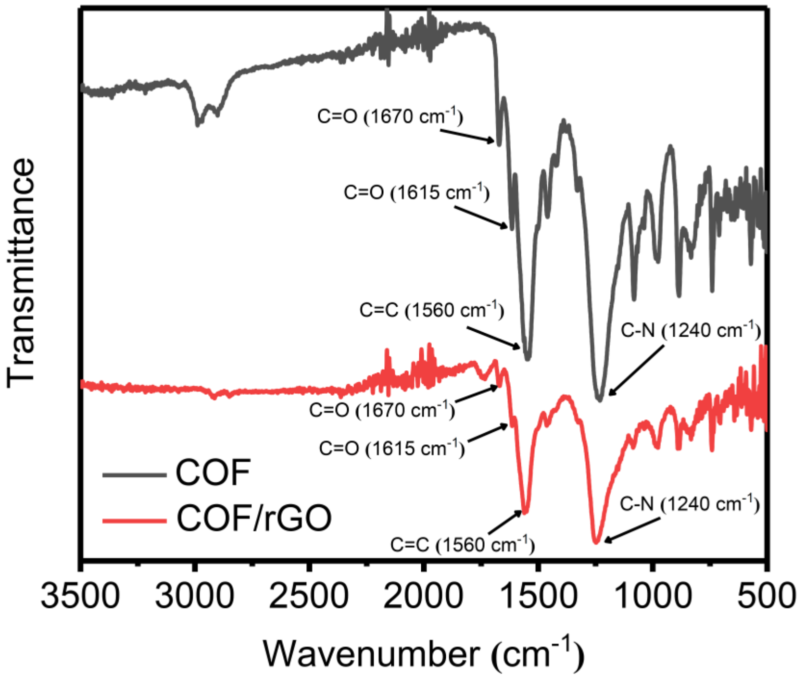

Supplementary Figure 10. IR spectra of COF and COF/rGO.

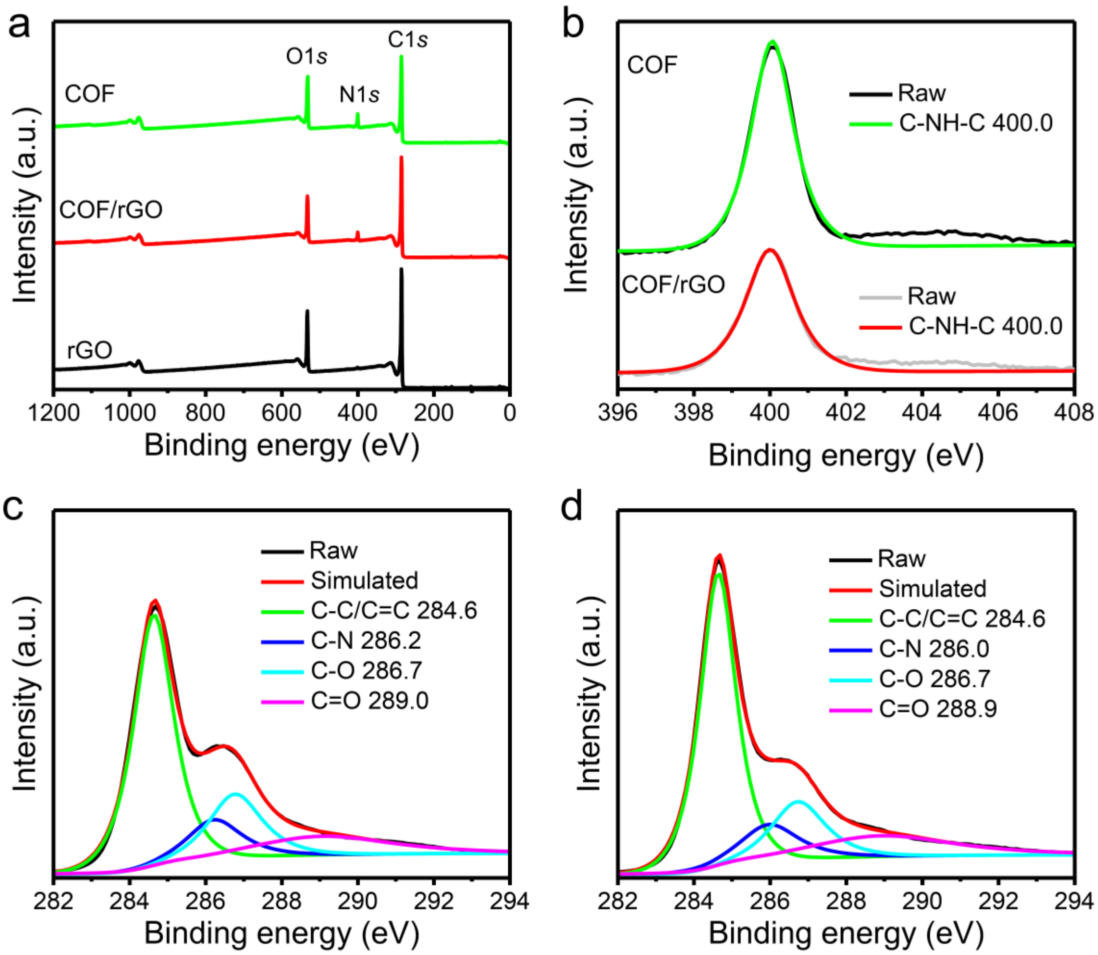

Supplementary Figure 11. (a) XPS survey spectra of COF, COF/rGO and rGO. (b) N1s XPS spectra of COF and COF/rGO. C1s XPS spectra of (c) COF and (d) COF/rGO.

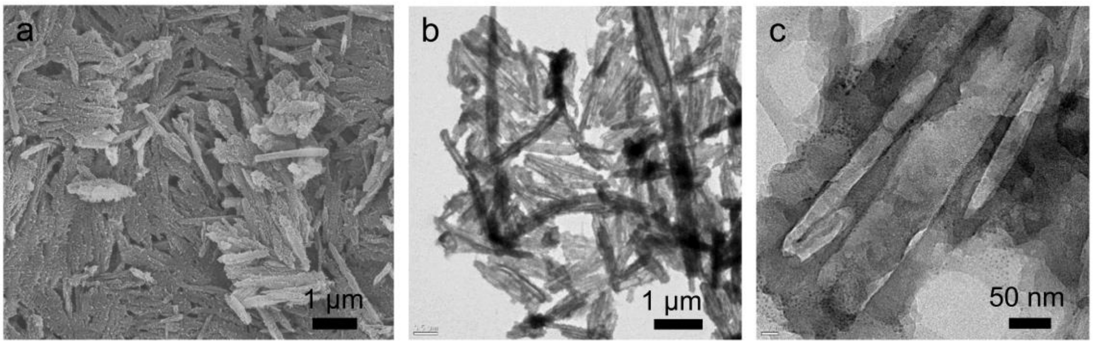

Supplementary Figure 12. (a) SEM and (b,c) TEM images of TpDq-COF.

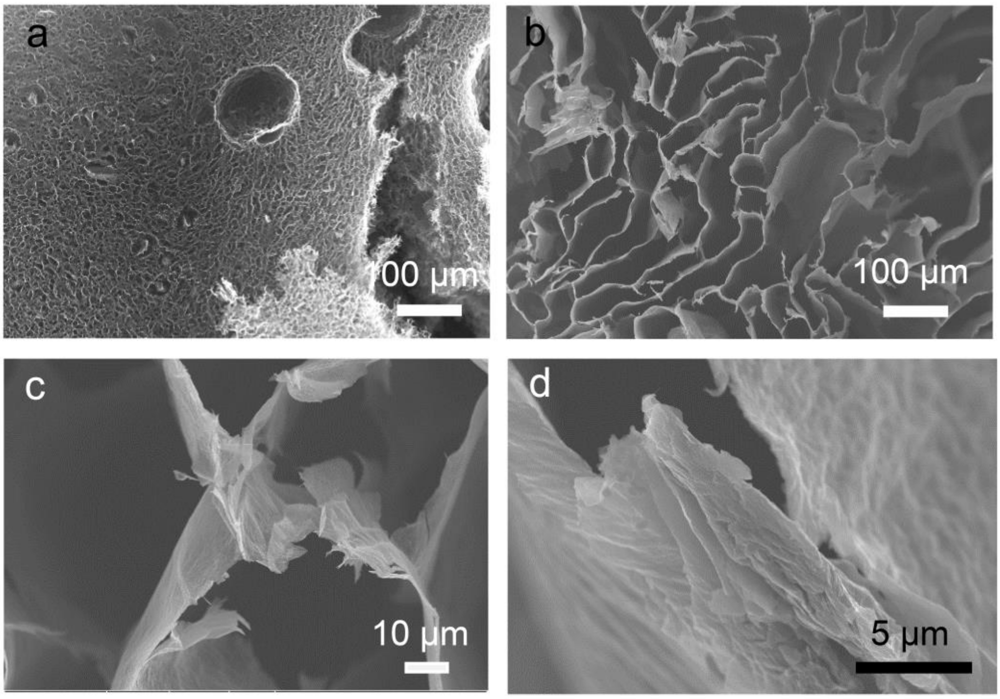

Supplementary Figure 13. SEM images of (a) COF/rGO aerogel, and (b-d) rGO aerogel.

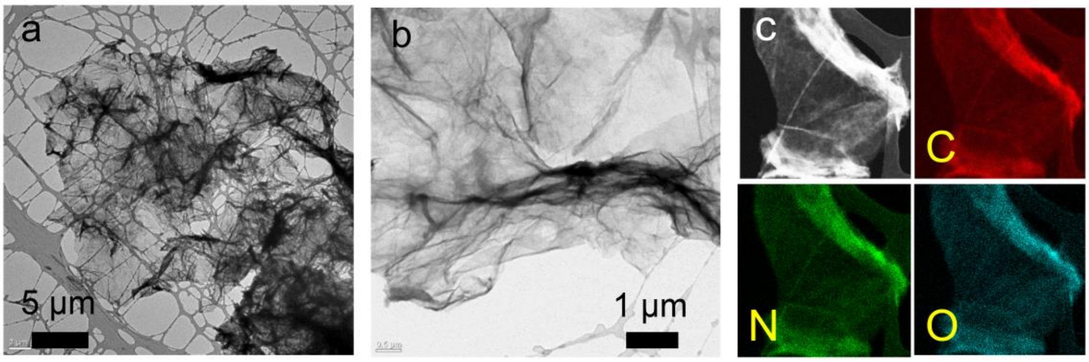

Supplementary Figure 14. (a,b) TEM image and (c) STEM-EDS elemental mapping images of COF/rGO.

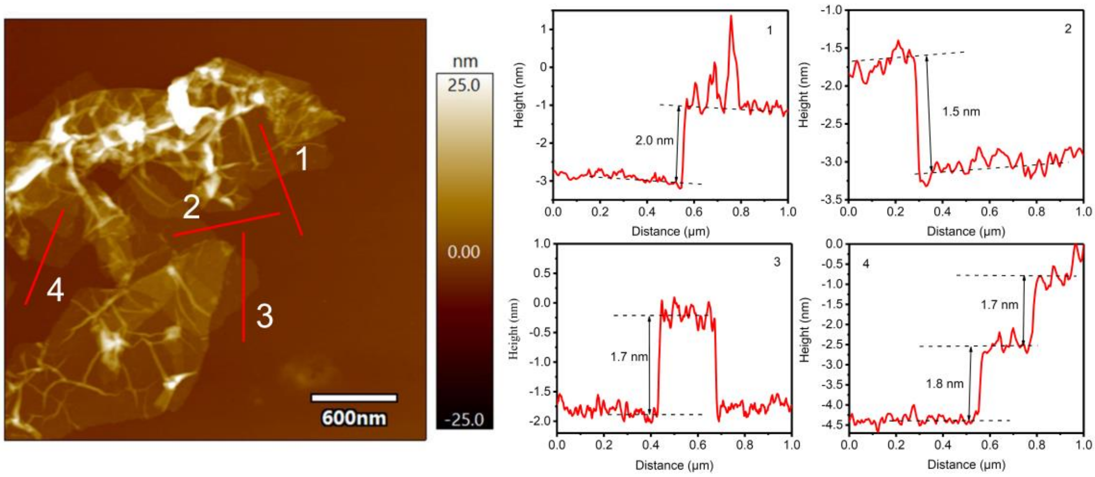

Supplementary Figure 15. AFM image and the corresponding height profiles for rGO.

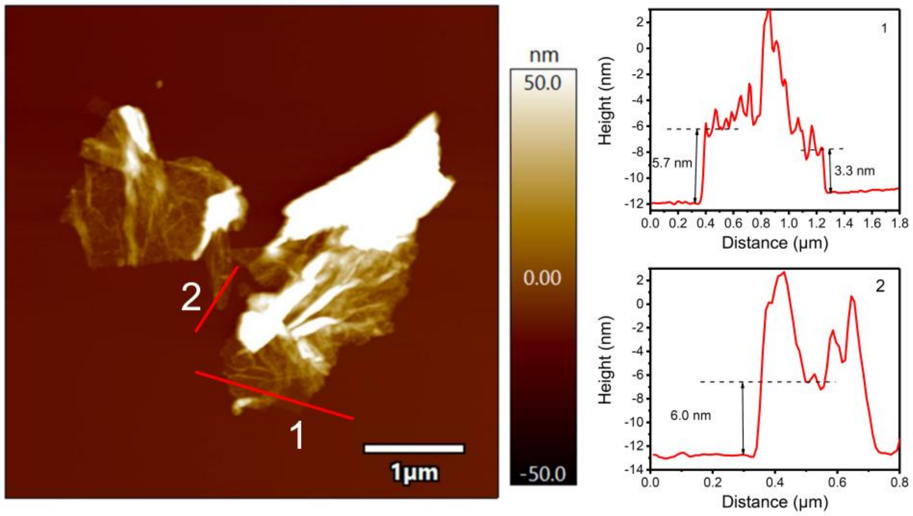

Supplementary Figure 16. AFM image and the corresponding height profiles for COF/rGO.

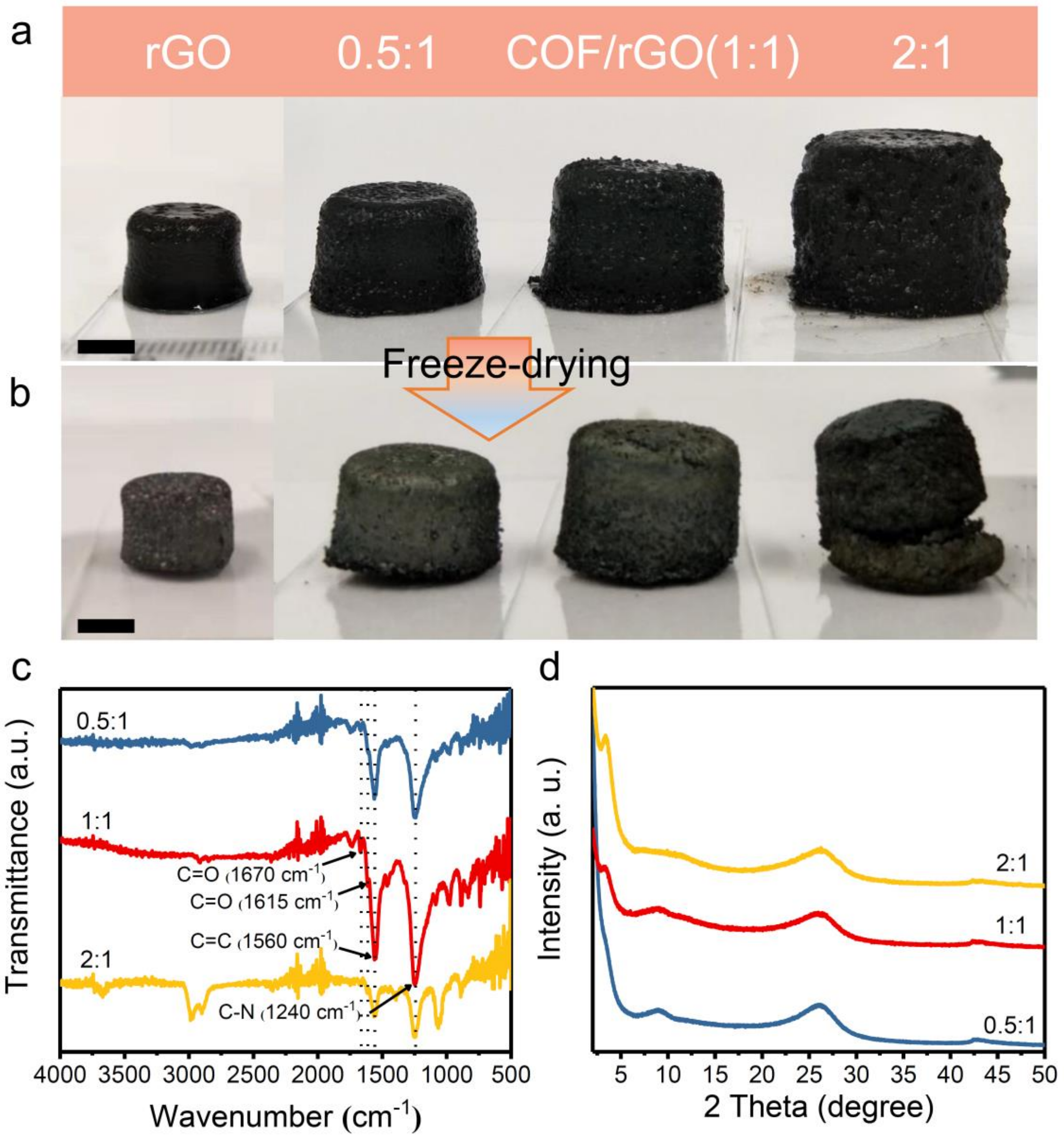

Supplementary Figure 17. 3D hydrogels and aerogels prepared from different ratios of COF monomers and GO. Photographs of (a) hydrogels of rGO and COF/rGOs and (b) the corresponding aerogels after freeze-drying. Scale bar: 0.5 cm. (c) IR spectra and (d) XRD patterns of COF/rGOs with different COF amounts.

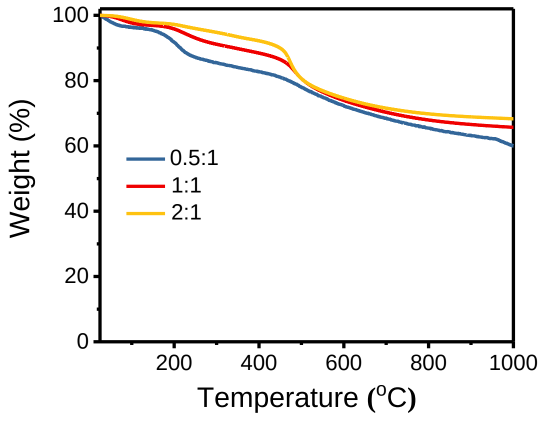

Supplementary Figure 18. TGA curves of COF/rGO hybrids with different amounts of COF.

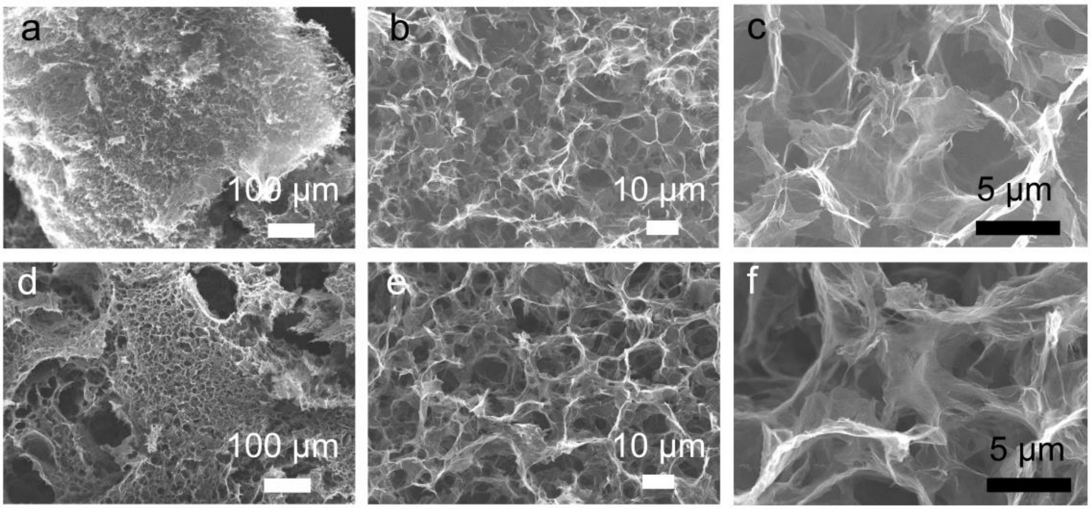

Supplementary Figure 19. SEM images of COF/rGO aerogels with different amounts of COF: (a–c) 0.5:1, and (d–f) 2:1.

Supplementary Note 3

The SEM images show the change of morphology with the introduction of different amounts of the COF. As shown in Supplementary Fig. 13b–d, the original rGO aerogel has a pore size of several hundred micrometers and very thick pore walls, from the stacking of the rGO layers. At a monomer:GO weight ratio of 0.5:1

(Supplementary Fig. 19a–c) the pore walls are much thinner, thus the formed COF on the rGO sheets is prevent GO from pronounced stacking during the hydrothermal process. However, when too much COF is introduced cross-linking of GO sheets is disturbed leading to partial fracture of the aerogel after freeze-drying (Supplementary Fig. 17b).

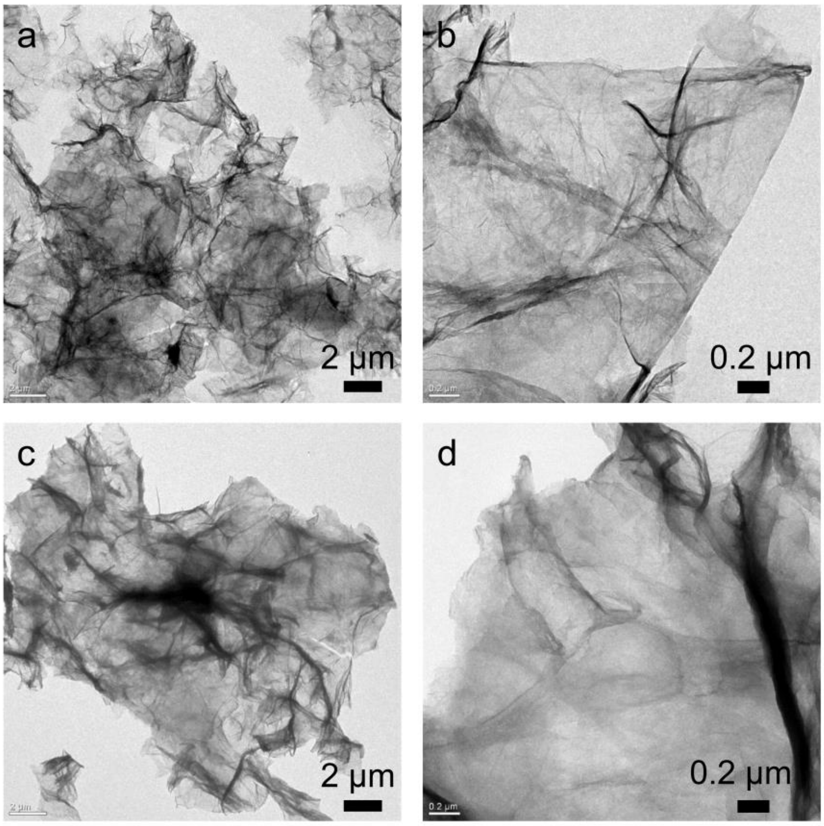

Supplementary Figure 20. TEM images of COF/rGO hybrids with different amounts of COF: (a-b) 0.5:1, and (c-d) 2:1.

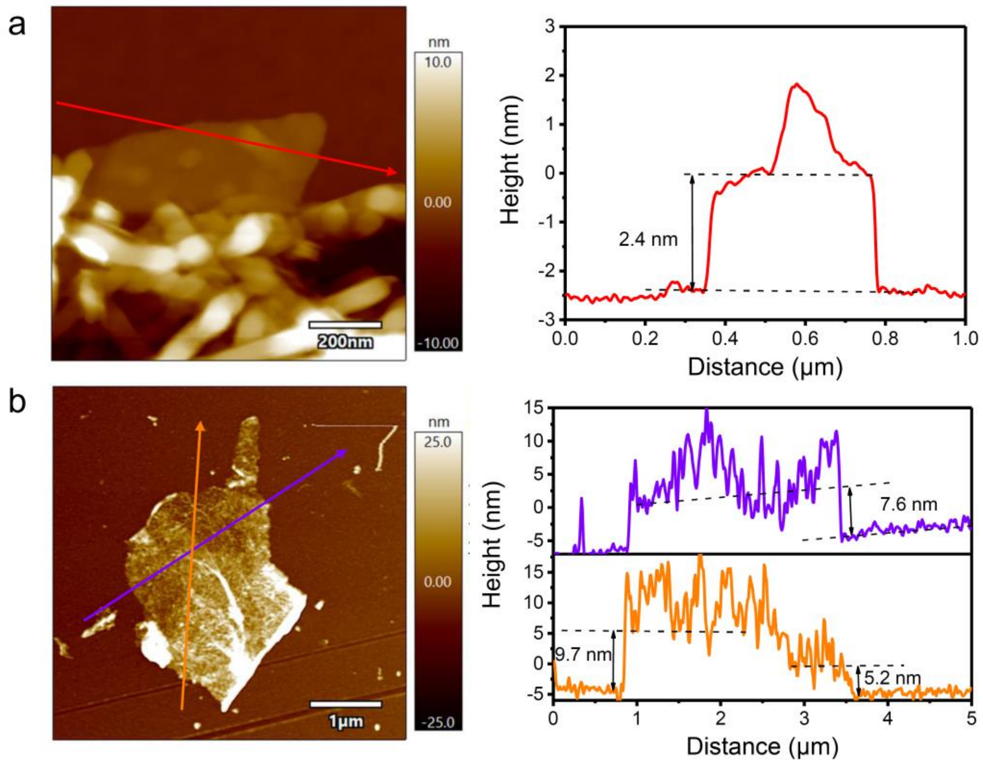

Supplementary Figure 21. AFM images and the corresponding height profiles for COF/rGO (a) 0.5:1 and (b) 2:1.

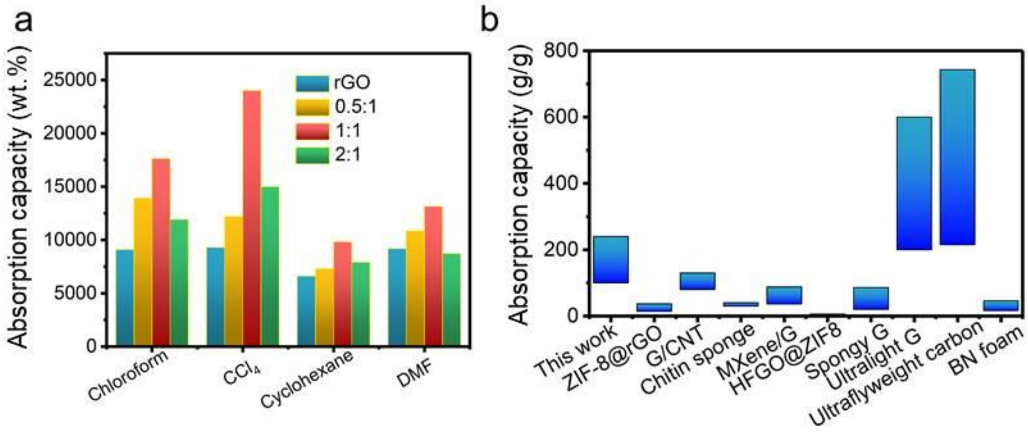

Supplementary Figure 22. (a) Absorption efficiency of rGO and COF/rGO hybrids with different amounts of COF. (b) Comparison of absorption capacities of different graphene-based or carbon-based materials.

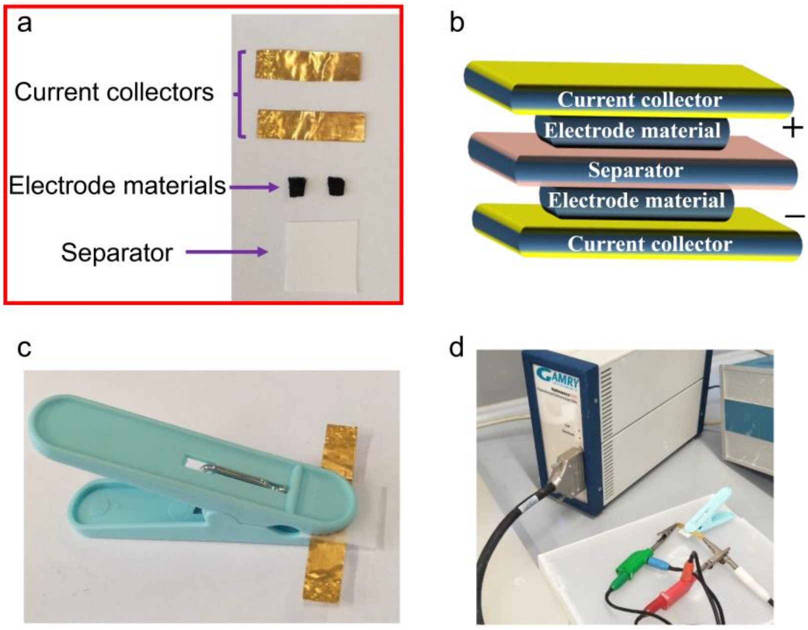

Supplementary Figure 23. A two-electrode symmetrical supercapacitor assembly. A filter paper separator soaked with 0.5 M H_2SO_4 aqueous electrolyte was used as separator and Au plates were used as current collectors. The whole device is fixed with a clip and wrapped with cling film to prevent the electrolyte from volatilizing.

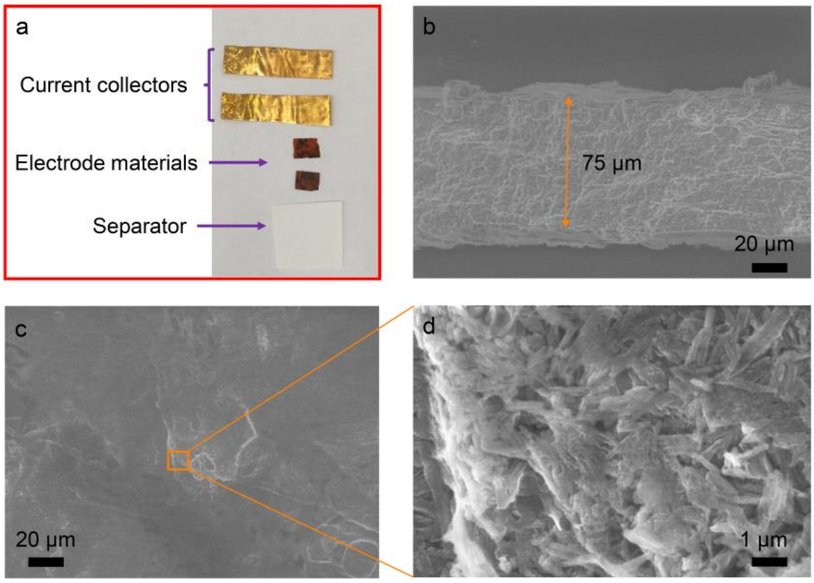

Supplementary Figure 24. (a) A two-electrode symmetrical supercapacitor assembly prepared with the pure TpDq-COF as electrode. (b) Cross section and (c, d) surface

SEM images of the COF electrode.

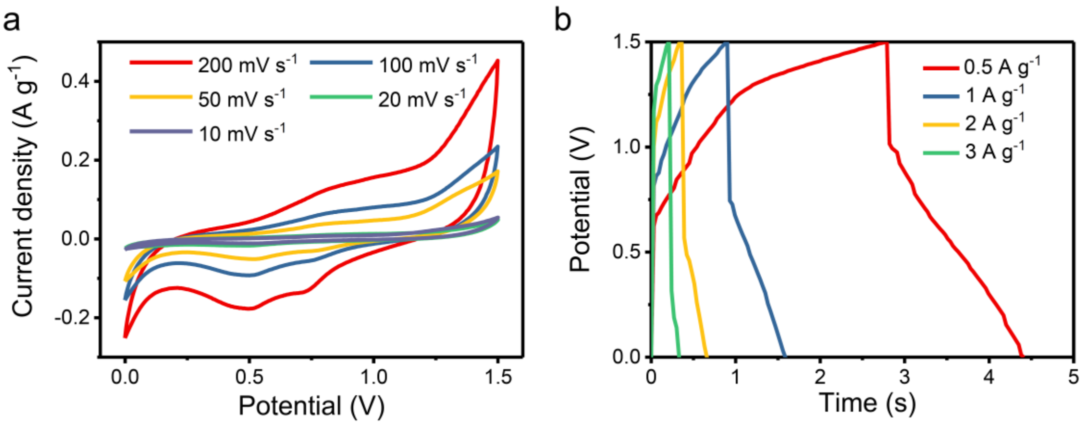

Supplementary Figure 25. (a) CV curves and (b) galvanostatic charge-discharge curves of TpDq-COF.

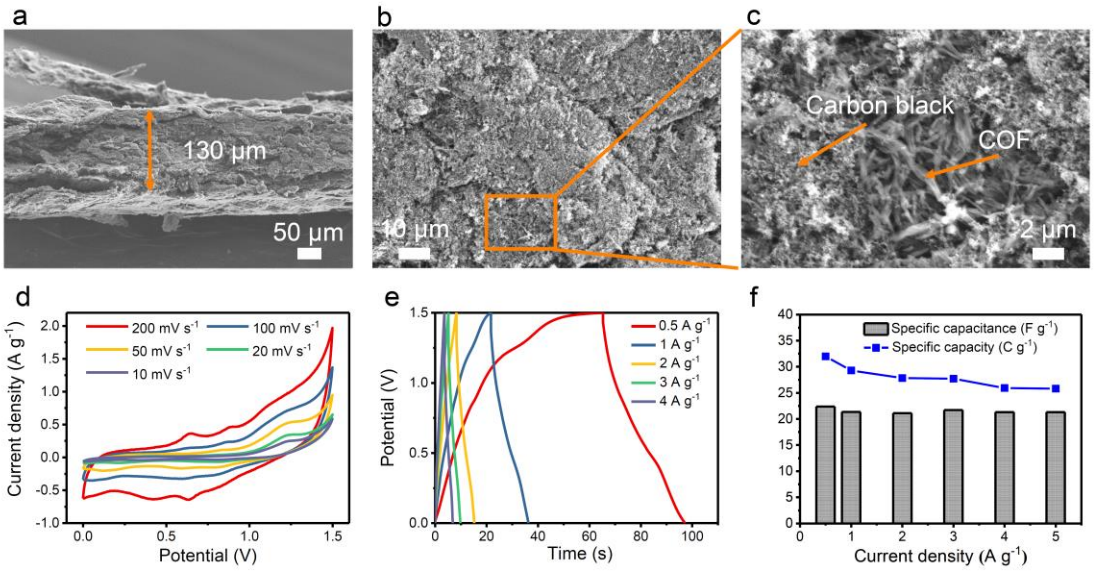

Supplementary Figure 26. (a) Cross section, and (b, c) surface SEM images of the COF-C-mix electrode. (d) CV curves and (e) the galvanostatic charge-discharge curves of COF-C-mix. (f) The specific capacitance and specific capacity of COF-C-mix calculated from the discharge curves under different current density.

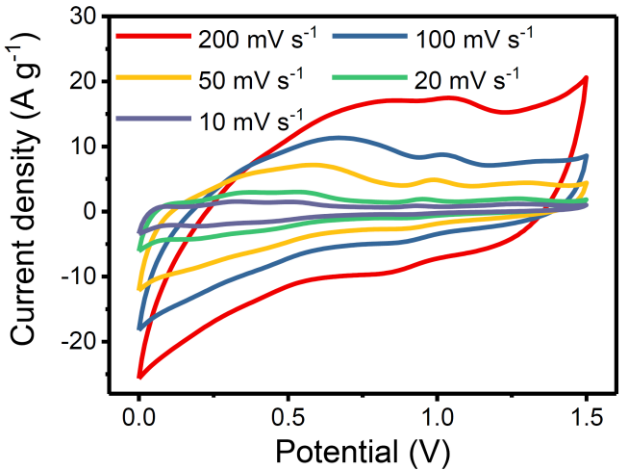

Supplementary Figure 27. CV curves of COF/rGO.

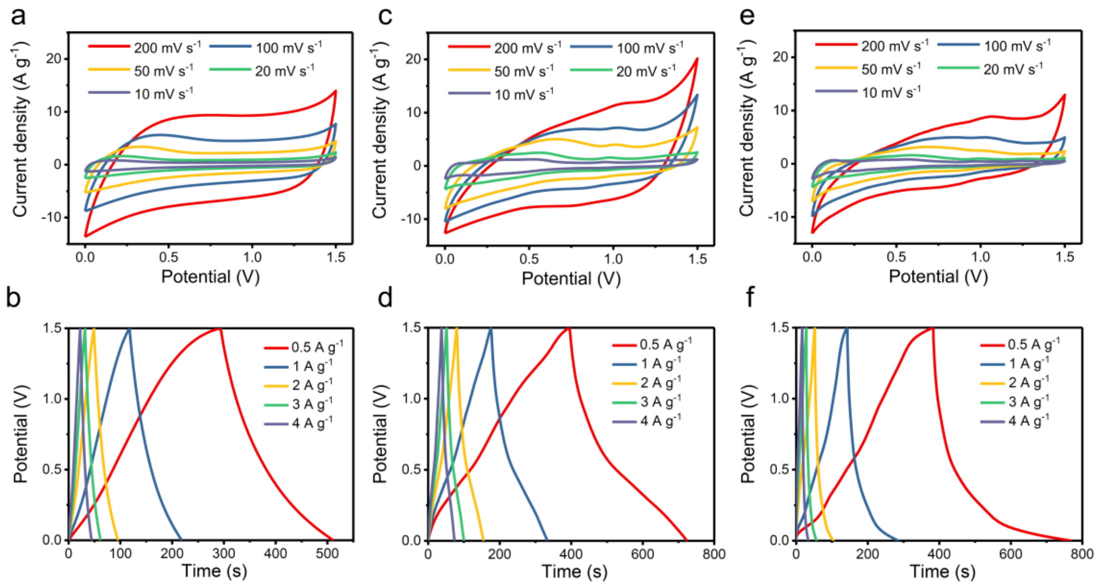

Supplementary Figure 28. CV and galvanostatic charge-discharge curves of (a, b) rGO, (c, d) 0.5:1 and (e, f) 2:1

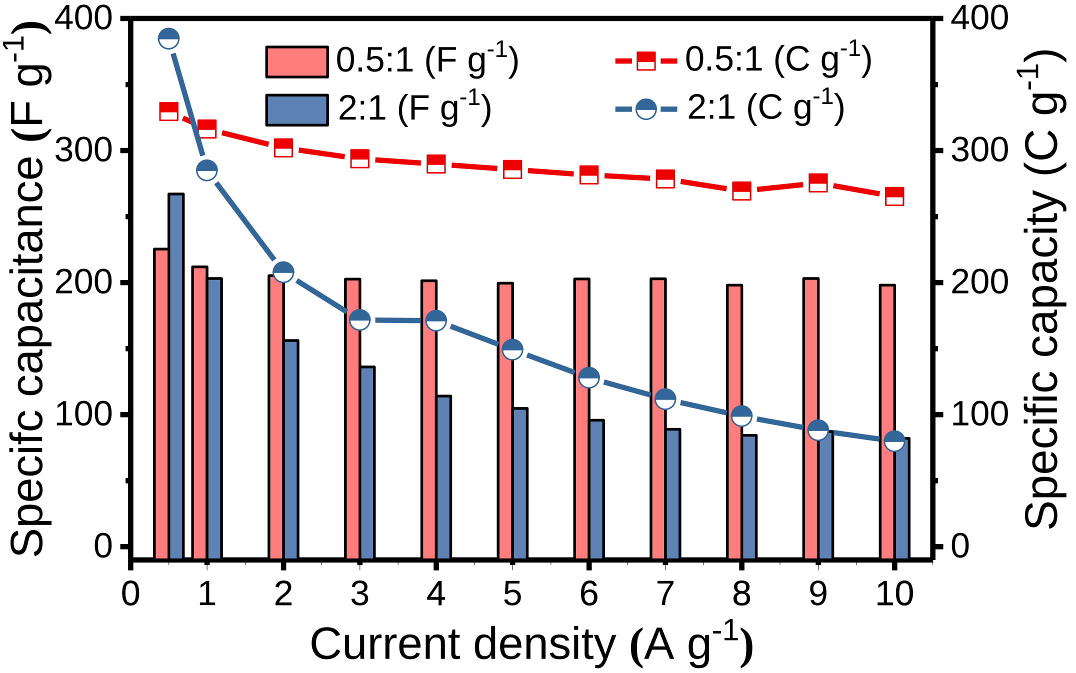

Supplementary Figure 29. The specific capacitance and specific capacity of COF/rGO hybrids (0.5:1 and 2:1) calculated from the discharge curves under different current density.

Supplementary Table 1. Density and elemental analysis COF/rGO aerogels.

Samples	Density (mg cm^{-3})	Cwt%	Hwt%	Nwt%
rGO	13±1.8	67.88	0.95	0.07*
0.5:1	7.5±0.4	69.82	2.05	2.42
1:1	7.0±0.5	69.11	2.06	4.52
2:1	9.0±0.8	67.32	2.3	4.9
COF	-	64.12	3.2	7.72

* Lower than the detection limit of the instrument.

The COF loading (wt%) in COF/rGO composites can be evaluated from the measured nitrogen content.

For 0.5:1, COF% = N% (0.5:1)/ N% (COF) = 2.42/7.72 × 100% = 31.3%.

For 1:1, COF% = N% (1:1)/ N% (COF) = 4.52/7.72 × 100% = 58.5%.

For 2:1, COF% = N% (2:1)/ N% (COF) = 4.9/7.72 × 100% = 64.0%.

Supplementary Table 2. Absorption capacities and density of typical absorbents.

Materials	Density (mg cm^{-3})	Absorbate	Capacity (g g^{-1})	Reference
COF/rGO	7.0±0.5	Hexane, Cyclohexane, Methanol, Toluene, DMSO, Silicone oil, Chloroform, Phenixin, Ethyl acetate, Acetone, DMF, Ethylene glycol, Dioxane, Ethanol, DMA, THF	98–240	This work
ZIF-8@rGO @Sponge	-	n-Heptane, ethyl acetate, dibromoethane, butanone, acetone, toluene, tetrachloromethane, chloroform, silicone oil, bump oil, bean oil	14–37	*Angew. Chem. Int. Ed.* **58**, 5297–5301 (2019)
Graphene–CNT hybrid foam	6.92	Compressor oil, sesame oil, chloroform, dichlorobenzene, toluene, DMF	80–130	*Chem. Commun.* **48**, 10660–10662 (2012).
Chitin sponges	20.1	Hexane, Gasoline oil, Cyclohexane, Corn oil, Toluene, Silicone oil, Pump oil, Engine oil, Chloroform, Phenixin	30–40	*ACS Appl. Mater. Interfaces* **6**, 19933–19942 (2014)
MXene/G	27	Hexane, Octane, Decane, Cyclohexane, Ethyl acetate, Acetone. DMF, Ethylene glycol, DMSO, Pump oil, Dioxane, Toluene, Phenoxin, THF, Dodecane	36–88	*Angew. Chem. Int. Ed.* **58**, 5297–5301 (2019)
BN	30.4	Formamide, Hexane, DMF, Benzene, 1-Butanol, Ethanol, Acetone, Salad oil, Dioxane, Oleic acid, Pumping oil, Dibutylphthalate, Silicone oil, Chloroform	16–46	*ACS Nano* **11**, 558−568 (2017)
FGO@MOG	-	toluene, hexane, heptane, decane, octadecane, petroleum ether, crude oil, veg oil, carbon tetrachloride	3–5	*Adv. Mater.* **29**, 1605307 (2017)
Spongy graphene	12	Methanol, Ethanol, Acetone, THF, DMSO, Toluene, Soybean oil, Caster oil, Kerosene, Pump oil, Dodecane, Decane, Octane, Heptane, Hexane, Nitrobenzene, Chloroform, 1,2-dichlorobenzene,	20–86	*Adv. Funct. Mater.* **22**, 4421–4425 (2012)

		Ethylbenzene		
Ultralight graphene framework	2.1±0.3	Ethanol, Acetone, Phenoxin, Cyclohexane, Chlorobenzene, Olive oil, Tetrahydrofuran (THF), Methanol, Toluene, Dimethyl sulfoxide (DMSO), Chloroform, Nitrobenzene, Gasoline	200–600	*Angew. Chem. Int. Ed.* **51**, 11371–11375 (2012)
Ultra-flyweight carbon aerogel	1.4	Hexane, Ethanol, Crude oil, Toluene, Motor oil, Veg oil, Dioxane, Ionic liquid (1-butyl-3-methylimidazolium tetrafluoroborate), Chloroform, Phenixin	215–743	*Adv. Mater.* **25**, 2554–2560 (2013).
HFGO@ZIF-8	-	Vegetable oil, Decaoctane, silicone oil, coconut oil, petroleum ether, chloroform	1.5–6	*Angew. Chem. Int. Ed.* **55**, 1178–1182 (2016).
Fe_2O_3/C foam	8.9	crude oil, bean oil, lubricating oil, hexane, gasoline, diesel oil, octane, decane, dodecane	60–102.6	*ACS Nano* **7**, 6875–6883 (2013).

Supplementary Table 3. Comparison of specific capacitance and specific capacity for COF/rGO, rGO and COF-based electrode materials.

Current density ($A g^{-1}$)	COF/rGO		rGO		COF	
	$F g^{-1}$	$C g^{-1}$	$F g^{-1}$	$C g^{-1}$	$F g^{-1}$	$C g^{-1}$
0.5	269	404	147	219	2.4	1.6
1	258	379	137	201	1.8	1.4
2	245	355	128	187	2.4	1.2
3	239	343	124	180	2.9	0.7
4	237	335	122	176	3.6	0.5
5	232	326	119	170	0	0
10	222	292	113	149	0	0

Supplementary Table 4. Comparison of specific capacitance for different COFs based materials.

Materials	Potential window (V)	Capacitance (F g^{-1})	Retention	Ref.
COF/rGO	0–1.5	269 at 0.5 A g^{-1}	96% after 5000 cycles	Present work
PDC-MA-COF	0–1.5	94 at 1.0 A g^{-1}	88% after 20000 cycles	*ACS Appl. Mater. Interfaces* **11**, 26355–26363 (2019)
TpPa-$(OH)_2$	0–0.7	214 at 0.2 A g^{-1}	88% after 10000 cycles	*Chem. Mater.* **29**, 2074–2080 (2017)
DAAQ-TFP COF*	-0.3–0.3	48±10 at 10 mV s^{-1}	82% after 5000 cycles	*J. Am. Chem. Soc.* **135**, 16821–16824 (2013)
$[TEMPO]_{100\%}$-NiP-COF<br>$[TEMPO]_{50\%}$-NiP-COF	0–0.8	(i) 167 at 0.1 A g^{-1}<br>(ii) 124 0.1 A g^{-1}	(i) 81% after 2000 cycles<br>(ii) 70% after 2000 cycles	*Angew. Chem. Int. Ed.* **54**, 6814–6818 (2015)
Dq_1Da_1Tp COF<br>DqTp COF	0–1	111 at 1.56 mA cm^{-2}<br>154 at 1.56 mA cm^{-2}	90% after 2500 cycles;<br>80% after 2500 cycles	*ACS Appl. Mater. Interfaces* **10**, 28139–28146 (2018)
COF/NH_2-rGO*	0–0.5	533 at 0.2 A g^{-1}	79% after 1000 cycles	*RSC Adv.* **5**, 27290–27294 (2015)
TaPa-Py COF	0–0.8	102 at 0.5 A g^{-1}	92% after 6000 cycles	*J. Mater. Chem. A* **4**, 16312−16317 (2016)
TPT-DAHQ COF*	-1–0.5	256 at 0.5 A g^{-1}	98.8% after 1850 cycles	*Chem. Asian J.* **14**, 1429–1435 (2019)
TpOMe-DAQ*	-0.5–0.5	169 at 0.35 A g^{-1}	increment after 30000 cycles	*J. Am. Chem. Soc.* **140**, 10941–10945 (2018)
v-CNS-RGO	-0.5–0.5	ca. 165 at 0.5 A g^{-1}	ca. 100% after 3000 cycles	*Angew. Chem. Int. Ed.* **57**, 1034–1038 (2018)
Ni3(HITP)2*	0–1	111 at 0.05 A g^{-1}	90% after 10000 cycles	*Nat. Mater.* **16**, 220–224 (2017)

TPA–TPA-COF-1*	0.2–0.7	51.3 at 0.2 A g^{-1}	-	*J. Mater. Chem. A* **6**, 19532–19541 (2018)
TFP–NDA–COF*	0–1	379 at 2 mV s^{-1}	75% after 8000 cycles	*Microporous Mesoporous Mater.* **266**, 109–116 (2018)
$COF_{BTA\text{-}DPPD}$-rGO *	0–0.5	239.1 at 0.5 A g^{-1}	70% after 1500 cycles	*Microporous Mesoporous Mater.* **287**, 65–70 (2019)
AC//PG-BBT	0–1.5	220 at 1 A g^{-1}	-	*Polym. Chem.* **11**, 47–52 (2020)
Car-TPT COF*	0–0.6	17.4 at 0.2 A g^{-1}	-	*ACS Appl. Mater. Interfaces* **11**, 9343–9354 (2019)
NH_2-f-MWCNT @$COF_{TTA–DHTA}$	0–0.8	127.5 at 0.4 A g^{-1}	96% after 1000 cycles	*Chem. Commun.* **53**, 6303−6306 (2017)

*The specific capacitances were measured in three-electrode systems.

Supplementary Table 5. Comparison of specific capacitance of COF/rGO electrode with other reported material systems.

Materials	Potential window (V)	Capacitance (F g^{-1})	Retention	Ref.
COF/rGO	0–1.5	269 at 0.5 A g^{-1}	96% after 5000 cycles	Present work
Holey graphene hydrogels	0–1	283 at 1.0 A g^{-1}	ca. 94% after 20000 cycles	*Nano Lett.* **15**, 4605 (2015).
EGM-rGO	0–4	231 at 1 A g^{-1}	98.6% after 20000 cycles	*Nat Energy* **5,** 160–168 (2020)
MnO_2–CNT–graphene–Ni	-0.2–0.8	251 at 1 A g^{-1}	82% after 3000 cycles	*Nanoscale* **6**, 1079–1085 (2014)
rGO/MoO_3	0–1	404 at 0.5 A g^{-1}	ca. 80% after 5000 cycles	*Adv. Mater.* **27**, 4695–4701 (2015)
Co_3O_4/VAGN/CF	-0.5–0.5	580 at 1 A g^{-1}	86.3% after 20000 cycles	*ACS Nano* **9**, 5310–5317 (2015)
AC/MXene-2:1	0–2	126 at 0.1 A g^{-1}	92.4% after 100 000 cycles	*ACS Energy Lett.* **3**, 1597−1603 (2018)
β-$Co(OH)_2$//N-graphene	0–1.8	241.9 at 0.5 A g^{-1}	93.2% after 10000 cycles	*Angew. Chem. Int. Ed.* **53**, 12789–12793 (2014).
PPy nanoporous gold	0–0.85	270 at 0.6 A g^{-1}	-	*Adv. Mater.* **23**, 4098–4102 (2011)
Fe-based MOF/graphene aerogel*	0–1	353 at 20 A g^{-1}	74.4% after 10000 cycles	*Adv. Mater. Interfaces* **5**, 1701548 (2018)
Zn-Co-S*	0–0.55	1266 at 1 A g^{-1}	91% after 10000 cycles	*Angew. Chem. Int. Ed.* **56**, 7141–7145 (2017).
PANI/graphene*	0–0.8	719 at 1.4 A g^{-1}	91.3% after 10000 cycles	*Energy Environ. Sci.* **10**, 2372–2382 (2017)

*The specific capacitances were measured in three-electrode systems.

Supplementary References

1. Sui, Z. et al. Preparation of Three-Dimensional Graphene Oxide–Polyethylenimine Porous Materials as Dye and Gas Adsorbents. *ACS Appl. Mater. Interfaces* **5**, 18, 9172–9179 (2013).

2. Jakša, G. et al. XPS and AFM characterization of aminosilanes with different numbers of bonding sites on a silicon wafer. *Surf. Interface Anal.* **45**, 1709–1713 (2013).

3. Wu, Q. et al. High-performance supercapacitor electrodes based on graphene hydrogels modified with 2-aminoanthraquinone moieties. *Phys. Chem. Chem. Phys.* **13**, 11193–11198 (2011).
