# Supplementary material for: Ultralight covalent organic framework/graphene aerogels with hierarchical porosity
Source: Nat Commun. 2020 Sep 18;11:4712. doi: 10.1038/s41467-020-18427-3 (PMC7501297; doi:10.1038/s41467-020-18427-3)
Supplement: Supplementary file 3 — Description of Additional Supplementary Files [file 41467_2020_18427_MOESM3_ESM.pdf]

Description of Additional Supplementary Files

File Name: Supplementary Movie 1
Description: Movie showing that COF/rGO aerogel can completely spring back to its original shape after the stress is released

File Name: Supplementary Movie 2
Description: Movie showing that COF/rGO aerogel can separate chloroform (dyed with Oil Red) from water
